# Brief Report: The Go/No-Go Task Online: Inhibitory Control Deficits in Autism in a Large Sample

**DOI:** 10.1007/s10803-016-2788-3

**Published:** 2016-04-21

**Authors:** F. Uzefovsky, C. Allison, P. Smith, S. Baron-Cohen

**Affiliations:** Department of Psychiatry, Autism Research Centre, University of Cambridge, Douglas House, 18B Trumpington Road, Cambridge, CB2 8AH UK

**Keywords:** Inhibitory control, Autism, Go/No-Go, Autism Spectrum Quotient

## Abstract

Autism Spectrum Conditions (ASC, also referred to as Autism Spectrum Disorders) entail difficulties with inhibition: inhibiting action, inhibiting one’s own point of view, and inhibiting distractions that may interfere with a response set. However, the association between inhibitory control (IC) and ASC, especially in adulthood, is unclear. The current study measured IC, using the Go/No-Go task online, in a large adult sample of 201 people with ASC and 240 controls. Number of both False Alarm and False Positive responses were significantly associated with autistic traits and diagnostic status, separately, but not jointly. These findings suggest that deficits in inhibition are associated with ASC. Future studies need to investigate the role of inhibition in ASC in everyday difficulties.

## Introduction

Autism Spectrum Conditions (ASC) are a set of neurodevelopmental conditions defined by two classes of symptoms: difficulties with social communication, alongside unusually repetitive behaviors and narrow interests. We use the term ASC to refer to what the Diagnostic and Statistical Manual 5 (DSM-5; American Psychiatric Association 2013) refers to as Autism Spectrum Disorder (ASD), but opt for ‘condition’ rather than ‘disorder’ as the latter can be stigmatizing. Both classes of symptoms may in part involve difficulties in inhibitory control (IC), that is, the ability to inhibit a response that interferes with a cognitive goal (Rothbart and Posner 1985). In the social domain, deficits in theory of mind (ToM; Baron-Cohen et al. 2013) may be due to an inability to inhibit one’s own point of view, preventing one from considering that of another person (Carlson and Moses 2001). Unusually repetitive behaviors or narrow interests may be the result of a set of responses that are repeated without inhibition (Mosconi et al. 2009). ‘Conflict IC’, where the desired response conflicts with a prepotent response, is positively related to ToM in typically developing children (Carlson and Moses 2001; Carlson et al. 2002), and is impaired in children with ASC (Christ et al. 2007). Similarly, IC is associated with higher rates of repetitive behaviour in individuals with high-functioning autism (Mosconi et al. 2009). It is important to note that these are correlational studies so it is unknown if difficulties with ToM or repetitive behavior are caused by deficits in IC.

Others have challenged the notion that ToM deficits are secondary to executive dysfunction, and propose that ToM deficits are caused by atypical functioning of domain-specific neural circuits (Frith and Frith 1999). Equally, others have challenged the notion that unusually repetitive behaviour and narrow interests reflect deficits in inhibition and instead propose that this may reflect a cognitive style characterized by a strong systemizing drive (pattern-detection; Baron-Cohen 2006), where repetition is a positive trait because it reveals lawful regularities. Nevertheless, IC warrants investigation in its own right, particularly in light of the recent focus on an imbalance in the ratio of the inhibitory neurotransmitters GABA and Glutamate and the idea of an altered ratio of excitation and inhibition in ASC (Coghlan et al. 2012).

One of the most widely used measures of motor IC is the Go/No-Go task. In this task cues are presented to the participants so that for most of the trials a motor response is requested, thus creating a prepotent response. On fewer trials (the No-Go trials), a No-Go response is evoked, i.e. not doing anything. The inability to suppress the prepotent motor response in this task is referred to as a False Alarm (FA) response, denoting an error of commission or a failure in IC. A No-Go response on Go trials is a False Positive response and represents an error of omission, or an over-cautious approach. The No-Go response is associated with inhibition in the motor cortex (Waldvogel et al. 2000), and is affected by paired-pulse TMS which causes inhibition via GABAergic signaling (van den Wildenberg et al. 2010). In addition, a study that used proton magnetic resonance spectroscopy in adolescents and young adults suggests that higher accuracy in the No-Go trials (fewer false alarm errors) is associated with higher levels of GABA in the anterior cingulate cortex (Silveri et al. 2013).

Several studies have examined the association between performance on the GNG task and ASC, with mixed results. Some studies report differences between people with ASC and controls (Christ et al. 2007; Langen et al. 2012; Wilson et al. 2014; Xiao et al. 2012) while others have found no differences (Kana et al. 2007; Lee et al. 2009; Nydén et al. 1999; Ozonoff et al. 1994; Schmitz et al. 2006; Sinzig et al. 2008). These conflicting findings may be the result of a lack of power, as the samples used in many of these studies consisted of 10–18 participants per comparison group. A recent meta-analysis (Geurts et al. 2014) found that the ability for prepotent inhibition (accuracy and reaction time, measured with the GNG as well as other tasks) significantly differs between ASC and typical participants. In addition, most studies analyzed in this meta-analysis were conducted with children, as adult groups have rarely been tested. The meta-analysis (Geurts et al. 2014) revealed a significant moderating effect of age, such that differences in prepotent response inhibition between ASC and control groups decreased with age.

In the current study we aimed to examine performance on the Go/No-Go prepotent inhibition task in a large sample of adults with and without a diagnosis of ASC. We predicted that individuals with ASC would show more False Alarm (FA) and more False Positive (FP) errors than controls. In addition, and in line with the DSM-5 (American Psychiatric Association 2013) focus on descriptive dimensions versus categorical diagnoses, we examined the association between FA and FP errors and scores on the Autism Spectrum Quotient (AQ; Baron-Cohen et al. 2001), a continuous measure of autistic traits in the general and clinical populations.

## Methods

### Participants

695 adults (18 years and older) took part. Of those, 68 were excluded (27 from the ASC group and 41 from the control group) due to incomplete information in the GNG task (see description in the “Measures” section), resulting in a sample of 627 individuals: 213 high-functioning adults diagnosed with ASC (103 females; mean age = 37.14 ± 12.01) and 414 controls (285 females; mean age = 38.76 ± 13.07). The control group had substantially more female participants than did the ASC group. In order to create sex-balanced ASC and control groups, 122 of control women were randomly selected from the control group to be included in the analyses. In addition, participants’ scores on the AQ and on the Raven’s Advanced Progressive Matrices test (RAPM; Raven et al. 1994) were examined for outliers. Following this procedure, 14 participants (control N = 10, ASC N = 4) were removed from the analyses due to extremely low RAPM scores (all scored 0 %), and 2 participants were removed from the ASC group due to extremely low AQ scores (AQ = 14 and 16). The final sample consisted of 441 participants (control N = 240, 47.5 % females; ASC N = 201, 49.8 % females). In the final sample participant groups did not differ in the composition of sex, age and non-verbal IQ, as measured by an online RAPM. See Table 1 for details on sample composition.

**Table 1:** Sample composition

	ASC group [range]	Control group [range]	Significance
Sex	49.8 % females	47.5 % females	χ^2^ = .222, *p* = .64
Age	37.34 ± 11.95 [18–69]	38.22 ± 13.32 [18–79]	t_(437)_ = .733, *p* = .46
Non-verbal IQ (RAPM)	85.64 % ± 9.31 % [33.33–98.33 %]	86.77 % ± 8.10 % [34.72–98.33 %]	t_(439)_ = 1.58, *p* = .11
Autism Spectrum Quotient (AQ)	40.47 ± 5.09 [26–49]	21.20 ± 8.45 [4–47]	t_(401)_ = −29.50, *p* < .001

*ASC* Autism Spectrum Conditions, *RAPM* Raven Advanced Progressive Matrices

Participants were recruited through two separate websites. Participants with ASC were recruited through the Cambridge University Autism Research Centre website (www.autismresearchcentre.com), and were included in the ASC group only if they specifically indicated that they had been diagnosed with an ASC and provided information regarding the diagnosis (the name of the clinic where they were diagnosed, and the type of clinician conducting the diagnosis—psychiatrist, clinical psychologist, neurologist, or paediatrician). We consider these participants as ‘high-functioning’ because of their ability to sign up for on-line research and complete various questionnaires and tasks. Control participants were recruited via the Cambridge Psychology website (www.cambridgepsychology.com). This is a general psychology research website for individuals in the general population who want to take part in research. Participants were included in the control group if they indicated that they did not have a diagnosis of ASC, nor suspect they have ASC, nor have a family member with a diagnosis of ASC.

#### Ethical Approval

Informed consent was obtained from all individual participants. The study was approved by the Cambridge University Psychology Research Ethics Committee. All procedures performed were conducted in accordance with the ethical standards of the institutional and/or national research committee and with the 1964 Helsinki declaration and its later amendments or comparable ethical standards.

### Measures

#### Go/No-Go Task (GNG)

Participants completed the GNG online, on the same website they were recruited from. The task presentation and response recording were identical in every way for both websites. Participants were presented with the instructions to the task “If you see an arrow pointing left, click or press the left button. If you see an arrow pointing right, click or press the right button. If you see an arrow pointing up, don’t click or press any button.” After indicating that the participant understood the instructions, the task began. The GNG task consisted of 300 trials; 220 trials elicited a Go response (110 pressing the right button, 110 pressing the left button); and 80 trials (26.7 %) elicited a No-Go response (not pressing any button). Shockwave Flash in conjunction with ActionScript v1 were used to present the task. Before each trial a blank screen was presented for 100 ms, after which an arrow appeared on screen until a response was made and for up to 1200 ms. (recorded as a No-Go response). The arrow was 140 × 100 pixels, actual presentation size is dependent on device’ settings. No feedback was given upon response. Response time was recorded but not analyzed due to the unreliability of the recording procedure used. Participants were excluded from the analysis if they failed to respond correctly on at least 50 % of the Go-right or the Go-left trials, i.e. pressing the right arrow key on the Go-right trials and pressing the left arrow key on the Go-left trials (68 participants were excluded, see above). This stringent exclusion criterion (for each trial participants had the three possible responses: Go-right/Go-left/do not respond) was employed in order to make sure that only participants who were attending to the task were included in the analysis. A ‘Go’ response in a No-Go trial was counted as a false alarm (FA) response; A ‘No-Go’ response in a ‘Go’ trial was counted as a false positive (FP) response. For each participant the number of FA’s and FP’s was summed and transformed into Z-scores, due to the skewness of the raw-sums distributions (2.91 ± .11 and 2.86 ± .11, respectively; most participants had very few mistakes).

#### The Autism Spectrum Quotient (AQ; Baron-Cohen et al. 2001)

The AQ is a self-report questionnaire that can be used to assess autistic tendencies in the general and clinical populations. The questionnaire consists of 50 items on a 4-point Likert scale (1-definitely agree to 4-definitely disagree). On each item a person can score 2, 1, or 0, with a higher score reflecting higher autistic tendencies. A sum score of all the items was used in this analysis. The AQ is useful in discriminating between ASC and controls, with 79.3 % of adults with ASC and only 2 % of controls scoring above 32 (Baron-Cohen et al. 2001).

#### Raven’s Advanced Progressive Matrices (RAPM; Raven et al. 1994)

The RAPM is a measure of non-verbal IQ. Scores were calculated as percent of correct responses to items for which a response was entered. This online measure was used to make sure that the ASC and control groups were comparable in terms of non-verbal IQ.

## Results

### False Alarm Errors

We examined the association between number of False Alarm (FA) errors and diagnosis using a regression model in three steps. In the first step the control variables age and sex were entered. In the second step we examined the effect of autistic traits as measured by the AQ. In the final step diagnosis was entered as the predictor.

AQ score was significantly associated with FA (β = .13, *p* < .01), yet when diagnosis was entered into the model, neither AQ score nor diagnosis significantly predicted FA (*p* = .72 and *p* = .10, respectively), probably due to multicollinearity between AQ and diagnosis. See Table 2 for details of the full model.

**Table 2:** Predicting False Alarm errors-values are based on the full model

Predictors	Beta	R^2^ change	F change
Step 1			
Age	.084	.021	4.706
Gender	−.123*		
Step 2			
Autism Quotient	.028	.018	8.156
Step 3			
Diagnosis	.132	.006	2.801
*adjR* ^2^ Total .036, *F* (4,4436) = 5.15, *p* < .001			

Beta values are derived from the full model which includes: gender, age, Autism Spectrum Quotient score and diagnosis

* *p* < .05

In order to test this hypothesis we examined a model with age, sex and diagnosis as the predictors (i.e. removing AQ from the regression analysis). This resulted in a significant association between FA and diagnosis (β = .15, *p* = .001), suggesting that diagnostic status was non-significant in the previous model due to the high correlation between AQ and diagnostic status (Spearman rho = .809, *p* < .001). Interestingly, sex was a significant predictor of FA in the full model (*β* = −.12, *p* = .009) as well as in the alternative model (β = −.12, *p* = .009), with women on average making more FA mistakes than men. In order to better understand the association between autistic traits and FA, we analysed a similar regression model for the ASC and control groups separately. In both groups AQ score was not a significant predictor of FA (*p* = .663 and *p* = .498, respectively). Interestingly, sex was significantly associated with FA in ASC but not in the control group (*p* = .012 and *p* = .222, respectively).

### False Positive Errors

A similar analysis was conducted for FP errors. Autistic traits were significantly associated with FP (β = .13, *p* = .005), and this effect became non-significant once diagnosis was entered into the model (*p* = .53). Diagnosis was a non-significant predictor (β = .11, *p* = .18). Interestingly, age was a significant predictor of FP (β = .14, *p* = .003), with older participants making more mistakes. See Table 3.

**Table 3:** Predicting False Positive errors-values are based on the full model

Predictors	Beta	R^2^ change	F change
Step 1			
Age	.141*	.021	4.647
Sex	−.048		
Step 2			
Autism Spectrum Quotient	.049	.018	8.102
Step 3			
Diagnosis	.105	.004	1.768
*adjR* ^2^ Total .034, *F* (4,436) = 4.84, *p* < .001			

Beta values are derived from the full model which includes: gender, age, Autism Spectrum Quotient score and diagnosis

* *p* < .01

In order to understand the lack of significant association between diagnosis and FP errors, and whether it could be explained by the correlation between AQ scores and diagnosis, we again examined a model with age, sex, and diagnosis as the predictors (i.e. removing AQ from the model). This resulted in a significant association between FP and diagnosis (β = .15, *p* = .002), and between FP and age (β = .14, *p* = .002), suggesting that diagnostic status was not significant in the previous model due to the high correlation between AQ and diagnostic status. As with FA errors, we examined the association between autistic traits and FP errors for the ASC and control groups separately. In both groups AQ score was not a significant predictor of FP (*p* = .342 and *p* = .173, respectively). Importantly, FA and FP errors correlated (r = .628, *p* < .001), and this correlation was high and significant for both ASC and control groups (r = .611, *p* < .001 and r = .636, *p* < .001, respectively).

## Discussion

Using a large sample of high-functioning individuals diagnosed with ASC and controls, we found a significant effect of ASC on the number of FA and FP errors on the Go/No-Go task. For both FA and FP the AQ score and diagnosis captured much of the same variation, so that either AQ score or diagnosis were sufficient to predict the level of inhibition and over-cautious response tendencies.

A meta-analysis of the differences in prepotent response inhibition (FA errors) between individuals with ASC and controls, with a particular focus on children (as most studies have been conducted in children) showed that differences in response inhibition exist, reporting an effect size of b = .55, and these decrease with age (Geurts et al. 2014). This raises the question—do differences in inhibition persist with age? In the current study we find that although the effect size is smaller (Cohen’s d = .31, β = .15) for adults than that reported in the meta-analysis, there is nevertheless a significant effect in the adult population. Moreover, this difference is not moderated by age, suggesting that differences reach a plateau and persist into adulthood. Future studies should investigate the development of prepotent response inhibition, so as to gain a better understanding of possible critical periods for intervention. Importantly, the participants in the current study had a relatively high IQ and were high-functioning in terms of their ability to independently sign-up for research online, fill-out questionnaires and complete various tasks. A significant difference found based on this sample suggests that lower functioning individuals might have stronger impairments, and this needs to be investigated in future research. Interestingly, in the case of FP responses, there was an association with age, with older individuals making more FP errors. This suggests that FA and FP follow different lifetime developmental trajectories.

The current findings, in a large sample of adults, shed light on previous research. Kana et al. (2007) examined brain activation during a GNG task in adults with and without ASC (N = 12 in each group). Although no behavioural differences were found, the activity of brain areas associated with behavioural inhibition, such as the anterior cingulate cortex and the right insula, was reduced in individuals with ASC. Taken together with the current study, the findings suggest that the role of inhibition deficits in adult individuals with ASC remains significant even in adulthood, and additional studies into the neurobiology of the ‘inhibition network’ in adulthood are warranted.

The current study has several limitations. The ASC sample is not representative of the entire range of ASC. First, participants had a relatively high IQ; second, inclusion in the ASC group was based on a self-reported diagnosis that was only verified in a proportion of participants (those who have attended the CLASS clinic, or those who have participated in in-person testing using ADI-R and ADOS). We do not think this casts doubt on diagnosis as all individuals reporting a diagnosis of ASC also provided the name of the clinician who diagnosed them, and at which clinic, and we excluded anyone who was not diagnosed according to DSM-IV or 5 by a mental health professional. Self-reported diagnosis in online recruitment has been shown to be highly correlated to independently verified diagnosis (Daniels et al. 2012). Another limitation of the online data collection strategy was that response time (RT) could not be measured reliably. It is particularly important because differences between ASC and controls could be evident to a greater extent in RT than in the response, as RT is a more variable and more sensitive measure than a categorical response of act/inhibit action, especially as participants were given a fixed window of 1200 ms to respond. This question should be explored in future studies.

Third, as mentioned before, the size of the difference between the typical and ASC groups was not large. Taking into account that the current study focused on a specific sub-group of high-functioning ASC, this difference is nevertheless of interest. The importance of further research into inhibitory control in ASC is even more evident when considering the high comorbidity between ASC and Attention Deficit Hyperactivity Disorder (ADHD), which studies estimate to be 28.2–78 % (Lai and Baron-Cohen 2015; Lee and Ousley 2006; Simonoff et al. 2008). The current research draws attention to a deficit in inhibitory control, evident even in high functioning individuals with ASC. Thus, it raises several important questions for future research: How do inhibition deficits contribute to clinical impairment in everyday functioning? Do individuals with ASC and associated learning difficulties show greater deficits in inhibition than higher-functioning individuals? How does ADHD comorbidity influence clinical impairment? Further research into inhibition in ASC using large samples is needed in order to accurately measure and investigate the implications of inhibition deficits in ASC.
